# Next-generation sequencing improves precision medicine in hearing loss

**DOI:** 10.3389/fgene.2023.1264899

**Published:** 2023-09-22

**Authors:** T. Imizcoz, C. Prieto-Matos, R. Manrique-Huarte, D. Calavia, A. Huarte, P. C. Pruneda, G. R. Ordoñez, E. Cañada-Higueras, A. Patiño-García, G. Alkorta-Aranburu, M. Manrique Rodríguez

**Affiliations:** ^1^ CIMA LAB Diagnostics, University of Navarra, Pamplona, Spain; ^2^ Department of Otorhinolaryngology, University Clinic of Navarra, Pamplona, Spain; ^3^ Dreamgenics S.L., Oviedo, Spain; ^4^ Centro de Secuenciación NASERTIC, Pamplona, Spain; ^5^ Genologica, Málaga, Spain; ^6^ Department of Pediatrics and Medical Genomics Unit, University Clinic of Navarra, Pamplona, Spain

**Keywords:** hearing loss, diagnosis, NGS, gene panel, precision medicine

## Abstract

**Background:** An early etiological diagnosis of hearing loss positively impacts children’s quality of life including language and cognitive development. Even though hearing loss associates with extremely high genetic and allelic heterogeneity, several studies have proven that Next-Generation Sequencing (NGS)-based gene panel testing significantly reduces the time between onset and diagnosis.

**Methods:** In order to assess the clinical utility of our custom NGS GHELP panel, the prevalence of pathogenic single nucleotide variants, indels or copy number variants was assessed by sequencing 171 nuclear and 8 mitochondrial genes in 155 Spanish individuals with hearing loss.

**Results:** A genetic diagnosis of hearing loss was achieved in 34% (52/155) of the individuals (5 out of 52 were syndromic). Among the diagnosed cases, 87% (45/52) and 12% (6/52) associated with autosomal recessive and dominant inheritance patterns respectively; remarkably, 2% (1/52) associated with mitochondrial inheritance pattern. Although the most frequently mutated genes in this cohort were consistent with those described in the literature (*GJB2, OTOF* or *MYO7A*), causative variants in less frequent genes such as *TMC1*, *FGF3* or *mitCOX1* were also identified. Moreover, 5% of the diagnosed cases (3/52) were associated with pathogenic copy number variants.

**Conclusion:** The clinical utility of NGS panels that allows identification of different types of pathogenic variants–not only single nucleotide variants/indels in both nuclear and mitochondrial genes but also copy number variants–has been demonstrated to reduce the clinical diagnostic odyssey in hearing loss. Thus, clinical implementation of genomic strategies within the regular clinical practice, and, more significantly, within the newborn screening protocols, is warranted.

## 1 Introduction

Hearing loss, defined as any degree of loss on the ability to hear sounds at thresholds considered normal, is the most prevalent sensory disorder (Kochhar et al., 2007; Li et al., 2022). Furthermore, over 5% of the world population presents disabling hearing loss, defined as any hearing loss greater than 35 dB according to the World Health Organization (WHO) (World health organization, 2023) and in European countries, 1-2 per 1000 newborns have hearing loss (del Castillo et al., 2022).

Hearing loss profoundly affects children’s quality of life. Since language acquisition is delayed, behavioral problems may arise and poor academic performance is usually related to hearing impairment (Guo et al., 2020; Lieu et al., 2020). An early etiologic diagnosis of hearing loss allows early intervention, which positively impacts on language, cognitive, emotional and social development, amongst others (Li et al., 2022). Hearing loss can be present at birth (congenital hearing loss) or it can be acquired later in life. It is one of the most etiologically heterogeneous traits (Lieu et al., 2020; Li et al., 2022) that can be associated with both environmental and genetic factors. Certain environmental factors such as cytomegalovirus or other TORCHes infections (toxoplasmosis, syphilis or hepatitis B, rubella, cytomegalovirus, herpes simplex), postnatal infections, ototoxicity or prematurity can explain about 30% of hearing loss cases in certain countries (Kochhar et al., 2007; Korver et al., 2017; Lieu et al., 2020). However, over half of the cases of hearing loss are due to pathogenic sequence variants, especially on developed countries where other ototoxic factors like noise exposure and infections are better controlled. Genetic hearing loss can be further classified into syndromic hearing loss, in which organs other than the ear are compromised (30% of the whole), and non-syndromic hearing loss, which accounts for 70% of the cases in which hearing loss presents as an isolated feature (Kochhar et al., 2007; Li et al., 2022; del Castillo et al., 2022; Lieu et al., 2020; García-García et al., 2020). Therefore, not only does a diagnosis of genetic hearing loss provide invaluable information regarding etiology, recurrence risk, prognosis or most suitable rehabilitation options, but it also may reveal other organs to be surveilled in the context of syndromic hearing loss, and it may help relief guilt that some parents may present due to misinformation and uncertainty (García-García et al., 2020).

Interestingly, new genomic diagnostic tools based on Next-Generation Sequencing (NGS) promise identification of pathogenic sequence variants associated with hearing loss not only as part of newborn screening test, but also for patients with delayed-onset hearing loss. Given nowadays affordable cost of genomic tools, implementation of NGS approaches in hearing loss etiological diagnosis is warranted (Li et al., 2022; del Castillo et al., 2022; Cabanillas et al., 2018), yet it is not common practice in the Spanish public health system.

The international collaboration project GHELP has engaged eight institutions in three countries (France, Spain and Portugal) in order to develop an NGS- based custom diagnostic tool that allows the identification of any type of pathogenic sequence variant (single nucleotide variants, SNVs; insertion-deletion variants, insertion/deletion variants, indels; copy number variants, CNVs) in all nuclear and mitochondrial genes previously associated with hereditary hearing loss. The aim of this study was to assess the prevalence of clinically relevant sequence changes in hearing loss individuals in the Spanish population with clinical suspicion of genetic origin and endorse the utility of a custom gene panel in early diagnosis and precision medicine in hearing loss.

## 2 Materials and methods

### 2.1 Research context

This research is circumscribed within the GHELP project “Boosting innovation in the early detection of hearing loss in children within SUDOE space: towards a personalized medicine based on genomic diagnostic tools”, co-financed by the Interreg Sudoe Program (abbreviation of Cooperation Program Interreg V-B Southwest Europe) through the European Regional Development Fund (ERDF).

### 2.2 Patients and clinical information

A total of 155 Spanish individuals with hearing loss were recruited for this study. Two cohorts of patients were defined, with unilateral o bilateral permanent hearing loss, with known clinical features (retrospective cohort) and patients in whom hearing loss has been detected during neonatal screening program and confirmed by auditory brainstem response (ABR) in at least one ear (prospective cohort).

- Retrospective cohort (n = 134 patients)**:** the inclusion criteria were age of onset of the hearing loss between 0 and 18 years (prelingual, peri-lingual and post-lingual), presence of permanent sensorineural hearing loss (SNHL) either in the presence or absence of a clinically defined syndrome and availability of clinical data at presentation and follow-up.
- Prospective cohort (n = 21 patients): the inclusion criteria were age of onset between 0 and 3 months, presence of any type SNHL and failure in the second tier of hearing screening with OtoAcoustic Emissions (OAE) and ABR.

For both cohorts, clinical information included demographic data, personal (pre-, peri-, and postnatal history) and family history, alterations in other organs and systems, performance on the hearing screening, results of the hearing tests performed and associated exploratory findings. In addition, for the retrospective cohort, data about the type of treatment for hearing loss and the results obtained following such treatments were recorded.

The clinical data was incorporated into a database for statistical analysis using the SPSS Statistics v20 software and consisted of a total of 83 variables for the retrospective cohort and 62 variables for the prospective cohort (Supplementary Table S1).

Only one patient in this study had been previously tested for common mutations in *GJB2* and the m.1555A>G mitochondrial DNA mutation in *MT-RNR1* (0.6%, 1 out 155, #P48). The remaining 154 patients were not prescreened for any common hearing loss mutation.

The study (#2016.034) was approved by the Ethics Clinical and Research Committee of the University Clinic of Navarra.

### 2.3 NGS GHELP panel development, validation and analysis

Thorough review of the available literature and commercially available tests for hearing loss allowed for the identification of a total of 179 genes, 171 nuclear and 8 mitochondrial, that were clinically related to any type of hereditary hearing loss (syndromic and non-syndromic) regardless the pattern of inheritance. Every commercially available gene with a strong evidence of relevance for hearing loss was included in the design. Furthermore, other candidate genes were included based on their frequency among our target populations and additional mitochondrial genes were also included in the design and define the list of target genes in our in-house-developed NGS GHELP panel design (Table 1). Target region included the entire coding sequence of these 179 genes ±10 bp of flanking intronic regions.

**TABLE 1:** Genes related to hearing loss included in our custom NGS GHELP panel design.

*ABHD12* (NM_001042472)	*DIAPH1* (NM_005219)	*LRTOMT* (NM_001145308)	*PRPS1* (NM_002764)
*ACTB* (NM_001101)	*DIAPH3* (NM_001042517)	*MARVELD2* (NM_001038603)	*PTPN11* (NM_002834)
*ACTG1* (NM_001614)	*DNMT1* (NM_001130823)	*MASP1* (NM_139125)	*PTPRQ* (NM_001145026)
*ADGRV1* (NM_032119)	*ECHS1* (NM_004092)	*MCM2* (NM_004526)	*RAF1* (NM_002880)
*AIFM1* (NM_004208)	*EDN3* (NM_207034)	*MIR96* (NR_029512)	*RDX* (NM_002906)
*ALMS1* (NM_015120)	*EDNRB* (NM_000115)	*MITF* (NM_000248)	*RMND1* (NM_017909)
*ANKH* (NM_054027)	*ELMOD3*	*MSRB3* (NM_198080)	*S1PR2* (NM_004230.4)
*AP1S1* (NM_001283)	*EPS8* (NM_004447)	MT-CO1	*SERAC1* (NM_032861)
*ATP1A3* (NM_152296)	*EPS8L2* (NM_022772)	MT-RNR1	*SERPINB6* (NM_004568)
*ATP6V1B1* (NM_001692)	*ESPN* (NM_031475)	MT-TE	*SIX1* (NM_005982)
*BCAP31* (NM_001139441)	*ESRRB* (NM_004452)	MT-TH	*SIX5* (NM_175875)
*BCS1L* (NM_004328)	*EYA1* (NM_000503)	MT-TK	*SLC17A8* (NM_139319)
*BDP1* (NM_018429)	*EYA4* (NM_004100)	MT-TL1	*SLC19A2* (NM_006996)
*BRAF* (NM_004333)	*FGF3* (NM_005247)	MT-TS1	*SLC26A4* (NM_000441)
*BSND* (NM_057176)	*FGFR3* (NM_000142)	MT-TS2	*SLC26A5* (NM_198999)
*CABP2* (NM_016366)	*FOXI1* (NM_012188)	*MYH14* (NM_024729)	*SLC33A1* (NM_004733)
*CACNA1D* (NM_000720)	*FTO* (NM_001080432)	*MYH9* (NM_002473)	*SLC52A2* (NM_024531)
*CCDC50* (NM_178335)	*GATA3* (NM_001002295)	*MYO15A* (NM_016239)	*SLC52A3* (NM_033409)
*CD164* (NM_006016)	*GIPC3* (NM_133261)	*MYO3A* (NM_017433)	*SLITRK6* (NM_032229)
*CDC14A* (NM_033312)	*GJB2* (NM_004004)	*MYO6* (NM_004999)	*SMPX* (NM_014332)
*CDH23* (NM_022124)	*GJB3* (NM_024009)	*MYO7A* (NM_000260)	*SNAI2* (NM_003068)
*CEACAM16* (NM_001039213)	*GJB6* (NM_006783)	*NARS2* (NM_024678)	*SOX10* (NM_006941)
*CHD7* (NM_017780)	*GPSM2* (NM_013296)	*NDP* (NM_000266)	*SPATA5* (NM_145207)
*CIB2* (NM_006383)	*GRHL2* (NM_024915)	*NLRP3* (NM_004895)	*STRC* (NM_153700)
*CISD2* (NM_001008388)	*GRXCR1* (NM_001080476)	*OPA1* (NM_015560)	*SYNE4* (NM_001039876)
*CLCNKA* (NM_004070)	*GSDME* (NM_004403)	*OSBPL2* (NM_014835)	*TBC1D24* (NM_020705)
*CLCNKB* (NM_000085)	*HARS2* (NM_012208)	*OTOA* (NM_144672)	*TECTA* (NM_005422)
*CLDN14* (NM_144492)	*HGF* (NM_000601)	*OTOF* (NM_194248)	*TIMM8A* (NM_004085)
*CLIC5* (NM_016929.5)	*HOMER2* (NM_004839)	*OTOG* (NM_173591)	*TJP2* (NM_004817)
*CLPP* (NM_006012)	*HOXA1* (NM_005522)	*OTOGL* (NM_173591)	*TMC1* (NM_138691)
*CLRN1* (NM_174878)	*HOXB1* (NM_002144)	*P2RX2* (NM_174873)	*TMIE* (NM_147196)
*COCH* (NM_004086)	*HSD17B4* (NM_000414)	*PAX3* (NM_181457)	*TMPRSS3* (NM_024022)
*COL11A1* (NM_001854)	*ILDR1* (NM_001199799)	*PCDH15* (NM_033056)	*TMPRSS5* (NM_030770)
*COL11A2* (NM_080680)	*JAG1* (NM_000214.3)	*PDZD7* (NM_001195263)	*TPRN* (NM_001128228)
*COL2A1* (NM_001844)	*KARS1* (NM_001130089)	*PEX1* (NM_000466)	*TRIOBP* (NM_001039141)
*COL4A3* (NM_000091)	*KCNE1* (NM_000219)	*PEX2* (NM_000318)	*TSPEAR* (NM_144991)
*COL4A4* (NM_000092)	*KCNJ10* (NM_002241)	*PEX26* (NM_017929)	*USH1C* (NM_005709)
*COL4A5* (NM_000495)	*KCNQ1* (NM_000218)	*PEX3* (NM_003630)	*USH1G* (NM_173477)
*COL4A6* (NM_001847)	*KCNQ4* (NM_004700)	*PEX5* (NM_001131025)	*USH2A* (NM_206933)
*COL9A1* (NM_001851)	*KITLG* (NM_000899)	*PEX6* (NM_000287)	*WFS1* (NM_006005)
*CRYM* (NM_001888)	*LARS2* (NM_015340)	*PJVK* (NM_001042702)	*WHRN* (NM_015404)
*DCAF17* (NM_025000)	*LHFPL5* (NM_182548)	*PMP22* (NM_000304)	*XYLT2* (NM_022167)
*DCDC2* (NM_016356)	*LHX3* (NM_014564)	*PNPT1* (NM_033109)	*CATSPER2* (STRCdel support)
*DDX11* (NM_030653)	*LOXHD1* (NM_144612)	*POU3F4* (NM_000307)	*CRYL1* (GJB6del support)
*DIABLO* (NM_019887)	*LRP2* (NM_004525)	*POU4F3* (NM_002700)	

Briefly, DNA was isolated from peripheral blood or saliva using the Maxwell RSC Instrument (Promega) and characterized by Qubit (ThermoFisher). Target-region capture using Agilent SureSelect custom probes-based enrichment protocol prior to paired-end Illumina MiSeq sequencing (control software 2.6.2.1) was established as the preferred wet-lab methodology at CIMA LAB Diagnostics (Universidad de Navarra).

The secondary and tertiary bioinformatic analyses were performed by Dreamgenics S.L through the Genome One platform, certified with the IVD/CE mark for *in vitro* diagnostic medical devices in accordance with current legislation (Licence Number: 7157-PS). A custom pipeline was used for secondary analysis of raw FASTQ files resulting from sequencing. The pipeline integrates the following steps: data quality control assessment using FastQC software (Babraham Bioi nformatics, 2023); removal of adapters and trimming of low-quality sequences by Trimmomatic (Bolger et al., 2014); alignment to GRCh38 human reference genome with BWA-mem (Li and Durbin, 2010); sorted bam files generation with SAMtools (Li et al., 2009) and removal of optical and PCR duplicates with Sambamba (Tarasov et al., 2015); variant calling of SNVs/indels using a combination of VarScan 2 (Koboldt et al., 2012) and Dreamgenics proprietary variant calling algorithm. For potential CNV identification, the pipeline also has a specific module based on an adaptation of the exome2cnv algorithm (Valdés-Mas et al., 2012). It also includes the first steps of tertiary analysis through variant annotation of SNVs/indels as follows: sequence variants description following Human Genome Variation Society (HGVS) recommendations and based on RefSeq database; variant information from different population databases (dbSNP, 1000 Genomes, ESP6500, ExAC, gnomAD, mtDB), *in silico* functional impact prediction (dbNSFP, dbscSNV) and clinical related database (ClinVar); variants visualization through an online platform allowing easy filtering and variant prioritization. Sequence variants were named following Human Genome Variation Society (HGVS) recommendations.

The genetic variants identified were classified following the 2015 American College of Medical Genetics and Genomics (ACMG) recommendation guidelines (Richards et al., 2015). Variants classified as pathogenic, likely pathogenic, and variants of unknown significance with a minimum depth of 50x for SNVs/indels, or a Genome One Quality Score over 2 for CNVs (following Dreamgenics guidelines) met our threshold for reporting. Pathogenic and likely pathogenic variants, if possible, were confirmed using an alternative technique such as Sanger Sequencing for SNVs/indels or MLPA (Multiplex Ligation–dependent Probe Amplification) for CNVs.

## 3 Results

Patients included in either the retrospective or prospective cohorts were classified in three different groups according to the genetic findings identified by analysis with the NGS GHELP panel - Group 1: individuals with a confirmed genetic diagnosis; Group 2: individuals for whom a clear genetic diagnosis was not achieved but a potential genetic origin could still be suspected based on the genetic variants identified, and Group 3: individuals for whom a genetic cause of hearing loss was not found.

### 3.1 Group 1

**FIGURE 1:**
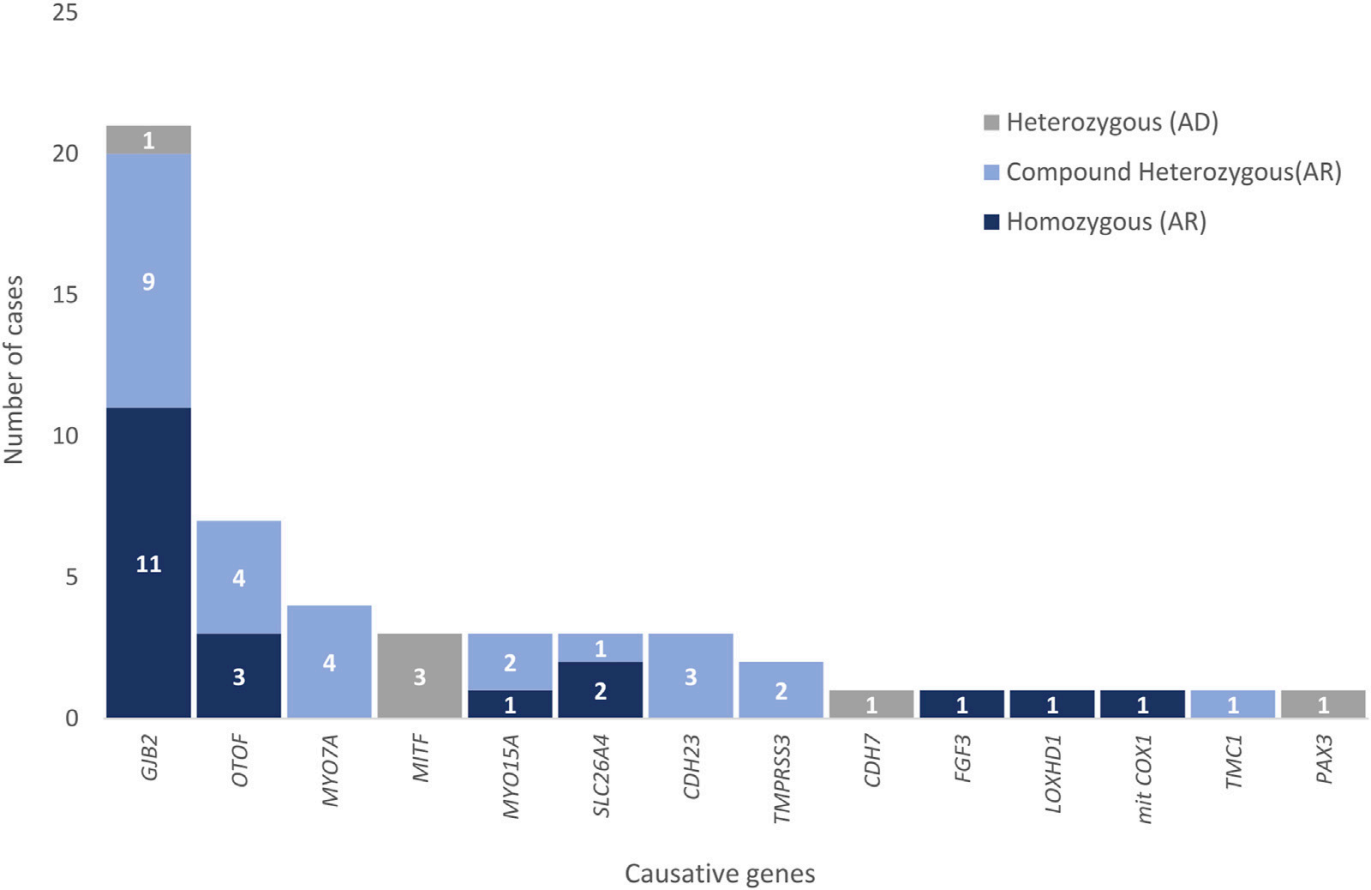
Causative genes responsible for hearing loss in patients in Group 1. Zygosity is indicated in different colors for each gene.

A genetic cause for hearing loss was identified in 32% of the patients (49 out of 155) (Table 2). The most frequently mutated genes in this cohort were consistent with those described in the literature (del Castillo et al., 2022; Lieu et al., 2020) being the most prevalent *GJB2* (n = 21)*, OTOF* (n = 7) and *MYO7A* (n = 4) (Figure 1). Summary of demographic and clinical traits of cases with a genetic diagnosis are displayed in Table 3.

**TABLE 2:** Pathogenic and likely pathogenic variants identified in patients from Group 1 for whom a genetic diagnosis for hearing loss was achieved. Variants indicated with the symbol ▲ are novel variants. (AR: autosomal recessive; AD: autosomal dominant; mit: mitochondrial inheritance; N/A not available; Comp. hetz: compound heterozygote; Homo: homozygote; Hetero: heterozygote; sdr.: syndrome).

Patient	Gene	Diagnosis	Inheritance	Zygosity	Mutation 1	Mutation 2
#P1	*GJB2*	DFNB1	AR	Comp hetz	c.35delG p.(Gly12Valfs*2)	c.227T>C p.(Leu76Pro)
#P2	*GJB2*	DFNB1	AR	Homo	c.35delG p.(Gly12Valfs*2)	N/A
#P3	*GJB2*	DFNB1	AR	Homo	c.35delG p.(Gly12Valfs*2)	N/A
#P4	*GJB2*	DFNB1	AR	Homo	c.101T>C p.(Met34Thr)	N/A
#P5	*GJB2*	DFNB1	AR	Homo	c.35delG p.(Gly12Valfs*2)	N/A
#P6	*GJB2*	DFNB1	AR	Homo	c.35delG p.(Gly12Valfs*2)	N/A
#P7	*GJB2*	DFNB1	AR	Comp hetz	c.617A>G p.(Asn206Ser)	c.109G>A p.(Val37Ile)
#P8	*GJB2*	DFNB1	AR	Comp hetz	c.35delG p.(Gly12Valfs*2)	c.139G>T p.(Glu47*)
#P9	*GJB2*	DFNB1	AR	Comp hetz	c.35delG p.(Gly12Valfs*2)	c.439G>A p.(Glu147Lys)
#P10	*GJB2*	DFNB1	AR	Homo	c.596C>T p.(Ser199Phe)	N/A
#P11	*GJB2*	DFNB1	AR	Homo	c.35delG p.(Gly12Valfs*2)	N/A
#P12	*GJB2*	DFNB1	AR	Homo	c.35delG p.(Gly12Valfs*2)	N/A
#P13	*GJB2*	DFNB1	AR	Comp hetz	c.35delG p.(Gly12Valfs*2)	c.101T>C p.(Met34Thr)
#P14	*GJB2/GJB6*	DFNB1	AR	Comp hetz	c.35delG p.(Gly12Valfs*2)	∆GJB6-D13S1830
#P15	*GJB2*	DFNB1	AR	Comp hetz	c.617A>G p.(Asn206Ser)	c.109G>A p.(Val37Ile)
#P16	*GJB2*	DFNB1	AR	Comp hetz	c.35delG p.(Gly12Valfs*2)	c.551G>C p.(Arg184Pro)
#P17	*GJB2*	DFNB1	AR	Comp hetz	c.35delG p.(Gly12Valfs*2)	c.227T>C p.(Leu76Pro)
#P18	*GJB2*	DFNB1	AR	Homo	c.35delG p.(Gly12Valfs*2)	N/A
#P19	*GJB2*	DFNA3	AD	Hetero	c.224G>A p.(Arg75Gln)	N/A
#P20	*GJB2*	DFNB1	AR	Homo	c.35delG p.(Gly12Valfs*2)	N/A
#P21	*GJB2*	DFNB1	AR	Homo	c.35delG p.(Gly12Valfs*2)	N/A
#P22	*OTOF*	DFNB9	AR	Homo	c.2485C>T p.(Gln829*)	N/A
#P23	*OTOF*	DFNB9	AR	Comp hetz	c.2485C>T p.(Gln829*)	c.5103 + 2T>A p.?
#P24	*OTOF*	DFNB9	AR	Comp hetz	c.2485C>T p.(Gln829*)	c.4275G>A p.(Trp1425*)
#P25	*OTOF*	DFNB9	AR	Homo	c.2485C>T p.(Gln829*)	N/A
#P26	*OTOF*	DFNB9	AR	Comp hetz	c.3002_3009del p.(Glu1001Valfs*18)▲	c.960 + 1G>T p.?▲
#P27	*OTOF*	DFNB9	AR	Comp hetz	c.2485C>T p.(Gln829*)	c.1236del p.(Glu413Asnfs*9)
#P28	*OTOF*	DFNB9	AR	Homo	c.2485C>T p.(Gln829*)	N/A
#P29	*MYO7A*	DFNA2/DFNA11/USH1B	AR	Comp hetz	c.397dup p.(His133Profs*7)	c.6431C>T p.(Thr2144Met)
#P30	*MYO7A*	DFNA2/DFNA11/USH1B	AR	Comp hetz	c.1996C>T p.(Arg666*)	c.4475C>T p.(Ala1492Val)
#P31	*MYO7A*	DFNA2/DFNA11/USH1B	AR	Comp hetz	c.397dupC p.(His133Profs*7)	c.6431C>T p.(Thr2144Met)
#P32	*MYO7A*	DFNA2/DFNA11/USH1B	AR	Comp hetz	c.6_9dup p.(Leu4Aspfs*39)▲	c.2116C>T p.(Gln706*)
#P33	*MITF*	Waanderburg sdr	AD	Hetero	Exons 2, 3, 4 and 5 deletion	N/A
#P34	*MITF*	Waanderburg sdr	AD	Hetero	Exons 2, 3, 4 and 5 deletion	N/A
#P35	*MITF*	Waanderburg sdr	AD	Hetero	c.640C>T p.(Arg214*)	N/A
#P36	*MYO15A*	DFNB3	AR	Comp hetz	c.3385C>T p.(Arg1129*)	c.9303 + 2T>G p.?▲
#P37	*MYO15A*	DFNB3	AR	Comp hetz	c.6004del p.(Glu2002Argfs*27)	c.8050T>C p.(Tyr2684His)
#P38	*MYO15A*	DFNB3	AR	Homo	c.6004del p.(Glu2002Argfs*27)	N/A
#P39	*SLC26A4*	Pendred sdr./DFNB4	AR	Comp hetz	c.416-1G>A p.?	c.1342-1G>A p.?
#P40	*SLC26A4*	Pendred sdr./DFNB4	AR	Homo	c.1198del p.(Cys400Valfs*32)	N/A
#P41	*SLC26A4*	Pendred sdr./DFNB4	AR	Homo	c.1963A>G p.(Ile655Val)	N/A
#P42	*TMPRSS3*	DFNB8	AR	Comp hetz	c.413C>A p.(Ala138Glu)	c.717C>A p.(Tyr239*)
#P43	*TMPRSS3*	DFNB8	AR	Comp hetz	c.413C>A p.(Ala138Glu)	c.717C>A p.(Tyr239*)
#P44	*CHD7*	CHARGE sdr	AD	Hetero	c.2959C>T p.(Arg987*)	N/A
#P45	*FGF3*	FGF3	AR	Homo	c.283C>T p.(Arg95Trp)	N/A
#P46	*LOXHD1*	DFNB77	AR	Homo	c.4480C>T p.(Arg1494*)	N/A
#P47	*mit COX1*	mit COX1	mit	MT_Homop	c.1542A>G p.(Term514Term)	N/A
#P48	*TMC1*	DFNB7	AR	Comp hetz	c.352G>T p.(Glu118*)	c.1404 + 1G>C p.?▲
#P49	*PAX3*	Waanderburg sdr	AD	Hetero	c.793-1G>A p.? ▲	N/A

**TABLE 3:** Summary of demographic and clinical traits of diagnosed cases in Group 1 regarding causative gene.

		Study Group		Family History		Gender		Onset		Audio profile			Progression		Vertigo/ tinnitus	
Causative Gene/ Diagnosis	N	Retros.	Pros.	No	Yes	Male	Female	Prel.	Postl.	P-SNHL	S-SNHL	Mo-SHL	No	Yes	No	Yes
*GJB2*	21	13	8	13	8	9	12	19	2	17	0	4	20	1	21	0
*OTOF*	7	5	2	4	3	2	5	7	0	7	0	0	4	3	7	0
*MITF*	3	3	0	0	3	0	3	3	0	2	1	0	3	0	3	0
*CDH23*	3	3	0	0	3	1	2	3	0	3	0	0	2	1	3	0
*PAX*	1	1	0	1	0	1	0	1	0	1	0	0	1	0	1	0
*MYO7A*	4	4	0	1	3	2	2	4	0	4	0	0	3	1	4	0
*MYO15A*	3	3	0	3	0	2	1	3	0	3	0	0	2	1	3	0
*SLC26A4*	3	3	0	2	1	2	1	3	0	3	0	0	2	1	3	0
*TMPRSS3*	2	2	0	2	0	1	1	0	2	2	0	0	0	2	2	0
*LOXHD1*	1	1	0	1	0	0	1	1	0	0	0	1	0	1	1	0
*TMC1*	1	1	0	0	1	0	1	1	0	1	0	0	1	0	1	0
*FGF3*	1	1	0	0	1	1	0	1	0	1	0	0	1	0	1	0
*COX1.null*	1	0	1	0	1	0	1	1	0	1	0	0	1	0	1	0
*CHD7*	1	1	0	1	0	0	1	1	0	1	0	0	1	0	1	0

Radiology							Treatment							Outcomes CAP**
							CI		HA		Bimodal	Bilateral ABI	BCI	
Causative Gene/ Diagnosis	Normal	SSCD	EVA	ICP	Michel aplasia	SHC	Bilat	Unilat	Bilat	Unilat				
*GJB2*	21	0	0	0	0	0	12	3	4	0	2	0	0	7.81
*OTOF*	7	0	0	0	0	0	6	1	0	0	0	0	0	7.66
*MITF*	3	0	0	0	0	0	2	0	0	1	0	0	0	7.66
*CDH23*	3	0	0	0	0	0	3	0	0	0	0	0	0	8.33
*PAX*	1	0	0	0	0	0	0	1	0	0	0	0	0	9
*MYO7A*	3	1	0	0	0	0	2	2	0	0	0	0	0	7.50
*MYO15A*	2	0	1	0	0	0	1	2	0	0	0	0	0	8.00
*SLC26A4*	0	0	3	0	0	0	1	2	0	0	0	0	0	7.00
*TMPRSS3*	2	0	0	0	0	0	2	0	0	0	0	0	0	7.00
*LOXHD1*	1	0	0	0	0	0	0	0	1	0	0	0	0	8.00
*TMC1*	0	0	0	1	0	0	1	0	0	0	0	0	0	8.00
*FGF3*	0	0	0	0	1	0	0	0	0	0	0	1	0	4.00
*COX1.null*	1	0	0	0	0	0	2	0	0	0	0	0	0	N/A
*CHD7*	0	0	0	0	0	1	1	0	0	0	1	0	1	6.5

Abbreviations Retros.: Retrospective; Pros.: Prospective; Prel.: Prelingual; Postl.: Postlingual; Bilat: bilateral; Unilat: unilateral; P-SNHL: profound sensorineural hearing loss; S-SNHL: severe sensorineural hearing loss; Mo-SHL: moderate sensorineural hearing loss; SSCD: Superior Semicircular Canal Dehiscence ; EVA: Enlarged Vestibular Aqueduct; SHC: Severe Hipoplasia Cochlea; ICP: Intracranial Pressure; ABI: Auditory brainstem implant; CI: Cochlear Implant; HA: Hearing Aid; BCI: Bone Conduction Implant; N/A: not available; CAP: Categories of Auditory Performance (CAP)-II. NEAP - Nottingham Early Assessment Package - © The Ear Foundation 2009

**CAP-II Categories. 0: No awareness of environmental sounds or voice. 1: Awareness of environmental sounds. 2: Response to speech sounds. 3: Identification of environmental sounds. 4: Discrimination of speech sounds without lip reading. 5: Understanding of common phrases without lip reading. 6: Understanding of conversation without lip reading. 7: Use of telephone with known speaker. 8: Follows group conversation in a reverberant room or where there is some interfering noise, such as a classroom or restaurant. 9: Use of telephone with an unknown speaker in unpredictable context.

**FIGURE 2:**
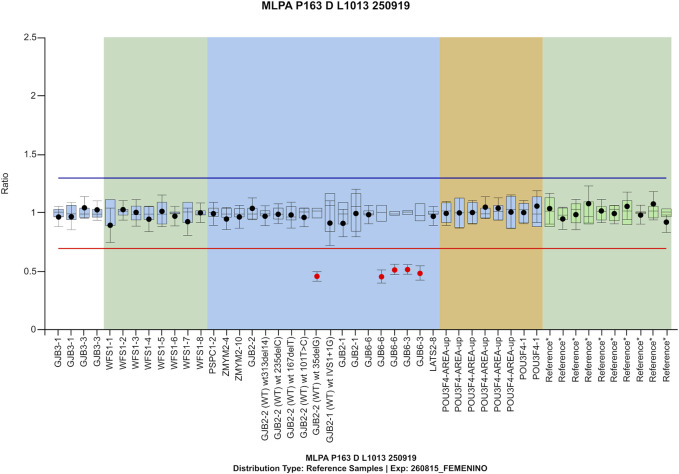
MLPA confirmation for compound heterozygosity/ digenic inheritance of GJB2 c.35delG and del(GJB6-D13S1830) in patient #P14 from family #F1.

Causative likely pathogenic/pathogenic variants were identified in genes associated with recessive SNHL in 86% of the cases (42 out of 49), in genes associated with autosomal dominant SNHL in 12% of the cases (6 out of 49), and in a mitochondrial gene in 2% of the cases (1 out of 49) in individuals in Group 1. The benefits of using a comprehensive genomic tool for the diagnosis of hearing loss extend beyond the identification of common single nucleotide variations in frequently mutated genes. Some of the advantages of using an NGS panel like ours that allows for the identification of any type of variant in either mitochondrial or nuclear genes for the diagnosis of hearing loss are exemplified by the following families:

Family #F1. A digenic inheritance of SNHL DFNB1 was identified in a 9-month-old patient with sensorineural profound bilateral hearing loss (#P14 in Table 2). The pathogenic genetic variants identified were *GJB2* (NM_004004.6): c.35delG p. (Gly12Valfs*2) and the well-described Spanish founder ∼309-Kb deletion ∆GJB6-D13S1830 (Del Castillo et al., 2003), which was validated by MLPA (Figure 2).

Family #F2. Two siblings (4 and 2 years old) presented with neurosensorial bilateral profound hearing loss with a family history of hearing impairment and clinical features of Waardenburg syndrome, including heterochromia iridis and presence of white forelock. In both cases, we identified a pathogenic heterozygous alteration consisting in the deletion of exons 2 to 5 of the *MITF* gene (#P33 y #P34 in Table 2). Heterozygous pathogenic or likely pathogenic alterations within this gene associate with Waardenburg syndrome type 2A, a dominant condition characterized by congenital hearing loss and pigmentary abnormalities of the hair, eyes and skin (Thongpradit et al., 2020).

Family #F3. A previously reported homplasmic pathogenic sequence change in the mitochondrial *mitCOX1* (also known as *MT-CO1* gene; NC_012920: m.7445A>G (Martin et al., 2000) was identified in a 1-year-old infant with sensorineural hearing loss, palmoplantar keratosis and other extra palmoplantar cutaneous features further described in an already published case report (Moreno-Artero et al., 2022) (#P47 in Table 2). Her mother also presented with severe-to-profound bilateral hearing loss since childhood, and she got cochlear implants in both ears. She was later studied and confirmed to be a carrier of the same mitochondrial variant.

Families #F4 and #F5. A 29-year-old patient in family #F4 presented with profound bilateral sensorineural hearing loss and good cochlear implant outcome in the right ear (#P19 in Table 2). No hearing device was used in the contralateral ear. Genetic testing identified the *GJB2* (NM_004004.6): c.224G>A p. (Arg75Gln) heterozygous pathogenic variant. This variant has already been described to act in a dominant manner causing non-syndromic hearing loss (DFNA3) or hearing loss with palmoplantar keratoderma (Feldmann et al., 2005). This subject did not present dermatological features, yet DFNA3 was diagnosed.

At the age of seven, a patient in family #F5 presented with moderate progressive bilateral sensorineural hearing loss. She also presented with erythematous plaques on the palms and a history of pruriginous red, dry skin patches (#P15 in Table 2). Previous negative genetic results for the most frequent mutations in both *GJB2* and *OTOF* were provided. However, we identified the presence of the following two pathogenic variants in *GJB2* (NM_004004.6): c.617A>G p. (Asn206Ser) and c.109G>A p. (Val37Ile). These two non-classical variants in *GJB2* have already been described as causative of non-syndromic hearing loss, ichthyosis and in some cases keratoderma on the palms and soles (Pang et al., 2014; Shen et al., 2019) which are consistent with the observed phenotype.

Besides the aforementioned families diagnosed with non-syndromic SNHL, five patients in this study (3%) were diagnosed with syndromic hearing loss, being *MITF* and *PAX3* pathogenic or likely pathogenic variants responsible of 4 cases of Waardenburg syndrome (#P33, #P34, #P35 and #P49 in Table 2). An additional patient was diagnosed with CHARGE syndrome (#P44 in Table 2). All the genetic findings in these patients were consistent with previously observed clinical features.

In addition, three patients (2%; #P39, #P40 and #P41 in Table 2) were found to be compound heterozygous or homozygous for variants in *SLC26A4,* related with either Pendred syndrome or autosomal recessive deafness-4 (DFNB4) with enlarged vestibular aqueduct, although the clinical signs usually associated with Pendred syndrome as temporal bone abnormalities and euthyroid goiter could not be clinically confirmed. Four additional individuals (3%; #P29, #P30, #P31 and #P32 in Table 2) were found to carry biallelic mutations in *MYO7A,* causative of either Usher syndrome type 1B or DFNA2/11, yet syndromic features related with Usher syndrome could not be demonstrated.

For patients in Group 1, segregation analyses in order to determine whether causative variants were in fact inherited in trans could not be performed in the context of this study. However, family segregation studies were recommended in the test report for every patient in Group 1 following European Society of Human Genetics (ESHG) recommendations for reporting results of diagnostic genetic testing (Deans et al., 2022).

### 3.2 Group 2

Individuals for whom a potential genetic cause could be suspected represents 25% (39 out of 155) of the subjects in the study. SNHL individuals were classified in this group 2 if 1) a pathogenic or likely pathogenic genetic variant and a variant of unknown clinical significance (VOUS) (Table 4), or two suspicious VOUS in genes associated with autosomal recessive SNHL or 2) a suspicious VOUS in a gene associated with autosomal dominant SNHL (Table 5) were identified. Any VOUS was considered suspicious if the observed clinical features were consistent with the described OMIM phenotype related to the gene in which the variant was identified. For genes inherited in an autosomal recessive manner, a VOUS was considered suspicious only when biallelic variants were identified. Follow-up functional or family member studies may help establish variant causality and therefore, determine whether there is actually a genetic cause underlying their SNHL.

**TABLE 4:** Patients from Group 2 in whom a pathogenic/likely pathogenic variant (variant 1) and a VOUS (Variant 2) were identified; a potential genetic cause could be suspected yet not confirmed. Variants indicated with the symbol ▲ are novel variants.

Patient	Gene	Variant 1	Variant 2
#P53	*MYO3A* (NM_017433)	c.3127del p.(Tyr1043Ilefs*6)▲	c.1828A>G p.(Ile610Val)
#P54	*MYO7A* (NM_000260)	c.640G>A p.(Gly214Arg)	c.323A>C p.(Tyr108Ser)▲
#P55	*GJB2* (NM_004004)	c.35delG p.(Gly12Valfs*2)	CRYL deletion
#P56	*NARS2* (NM_024678)	c.151C>T p.(Arg51Cys)	c.1403T>G p.(Phe468Cys)▲
#P57	*CDH23* (NM_022124)	c.4488G>G p.(Gln1496His)	c.1672G>A p.(Val558Met)
#P58	*MYO15A* (NM_016239)	c.4143-2_4143-1insT▲	c.9938A>G p.(His3313Arg)
#P59	*MYO6* (NM_004999)	c.3198del p.(Ala1068Glnfs*42)▲	c.3704G>T p.(Gly1235Val)
#P60	*CDH23* (NM_022124)	c.4844C>A p.(Ser1615*)▲	potential deletion, not confirmed
#P61	*USH1C* (NM_153676)	c.672C>A p.(Cys224*)	c.781G>T p.(Val261Phe)▲

**TABLE 5:** Patients from Group 2 in whom either two VOUS in AR genes or one VOUS in AD genes were identified; a potential genetic cause could be suspected yet not confirmed. Variants indicated with the symbol ▲ are novel variants.

Patient	Gene	Variant 1	Variant 2
#P62	*TMC1* (NM_138691)	c.309_310delinsGC p.(Ile103_Ala104delinsMetPro)▲	c.1679A>T p.(Asp560Val)▲
#P63	*MARVELD2* (NM_001038603)	c.1627A>T p.(Ile543Phe)▲	c.1627A>T p.(Ile543Phe)▲
#P64	*OTOF* (NM_194248.3)	c.154G>A p.(Val52Met)	c.1732G>C p.(Val578Leu)▲
#P65	*MYO15A* (NM_016239.4)	c.4072G>A p.(Gly1358Ser)	c.8315A>C p.(Tyr2772Ser)
#P66	*USH1C* (NM_153676)	c.940C>T p.(Arg314Trp)	c.1597G>A p.(Ala533Thr)
#P67	*SLC26A4* (NM_000441)	c.445G>A p.(Gly149Arg)	c.1370A>T p.(Asn457Ile)▲
#P68	*LOXHD1* (NM_144612.6)	c.4523G>A p.(Arg1508Lys)	c.4972C>A p.(Arg1658Ser)▲
#P69	*OTOG* (NM_001277269.2)	c.586G>A p.(Asp196Asn)	c.8161G>A p.(Asp2721Asn)
#P70	*PTPRQ* (NM_001145026.2)	c.937G>A p.(Val313Ile)	c.3148C>A p.(Gln1050Lys)
#P71	*EPS8* (NM_004447.6)	c.1627A>T p.(Ile543Phe)	potential deletion, not confirmed
#P72	*WHRN* (NM_015404.4)	c.1943C>A p.(Ser648Tyr)	c.2644C>A p.(Arg882Ser)
#P73	*ESRRB* (NM_004452.3)	c.746G>A p.(Arg249Gln)	c.1048G>A p.(Ala350Thr)
#P74	*TBC1D24* (NM_001199107.2)	c.641G>A p.(Arg214His)	c.641G>A p.(Arg214His)
#P75	*USH2A* (NM_206933.3)	c.5629G>C p.(Ala1877Pro)▲	c.8609C>T p.(Pro2870Leu)
#P76	*MITF* (NM_000248)	c.28T>A p.(Tyr10Asn)▲	N/A
#P77	*GSDME* (NM_004403.3)	c.712C>T p.(Arg238*)	N/A
#P78	*MCM2* (NM_004526.4)	c.2428G>A p.(Val810Ile)	N/A
#P79	*TECTA* (NM_005422.2)	c.2657A>G p.(Asn886Ser)	N/A
#P80	*COL11A2* (NM_080680.3)	c.353G>C p.(Arg118Pro)▲	N/A
#P81	*CHD7* (NM_017780.4)	c.6270G>T p.(Trp2090Cys)▲	N/A
#P82	*POU4F3* (NM_002700.3)	c.753delG p.(Trp251Cysfs*68)▲	N/A
#P83	*GATA3* (NM_001002295.2)	c.779-3_779-2delinsAG p.?▲	N/A
#P84	*WFS1* (NM_006005.3)	c.2405T>C p.(Ile802Thr)	N/A
#P85	*COL11A1* (NM_001854.4)	potential deletion, not confirmed	N/A
#P86	*KCNQ4* (NM_004700.4)	c.1723C>T p.(Arg575Trp)	N/A
#P87	*OSBPL2* (NM_144498.4)	c.1248G>A p.(Met416Ile)▲	N/A
#P88	*MYH14* (NM_024729.3)	c.3281G>A p.(Arg1094Gln)	N/A

**FIGURE 3:**
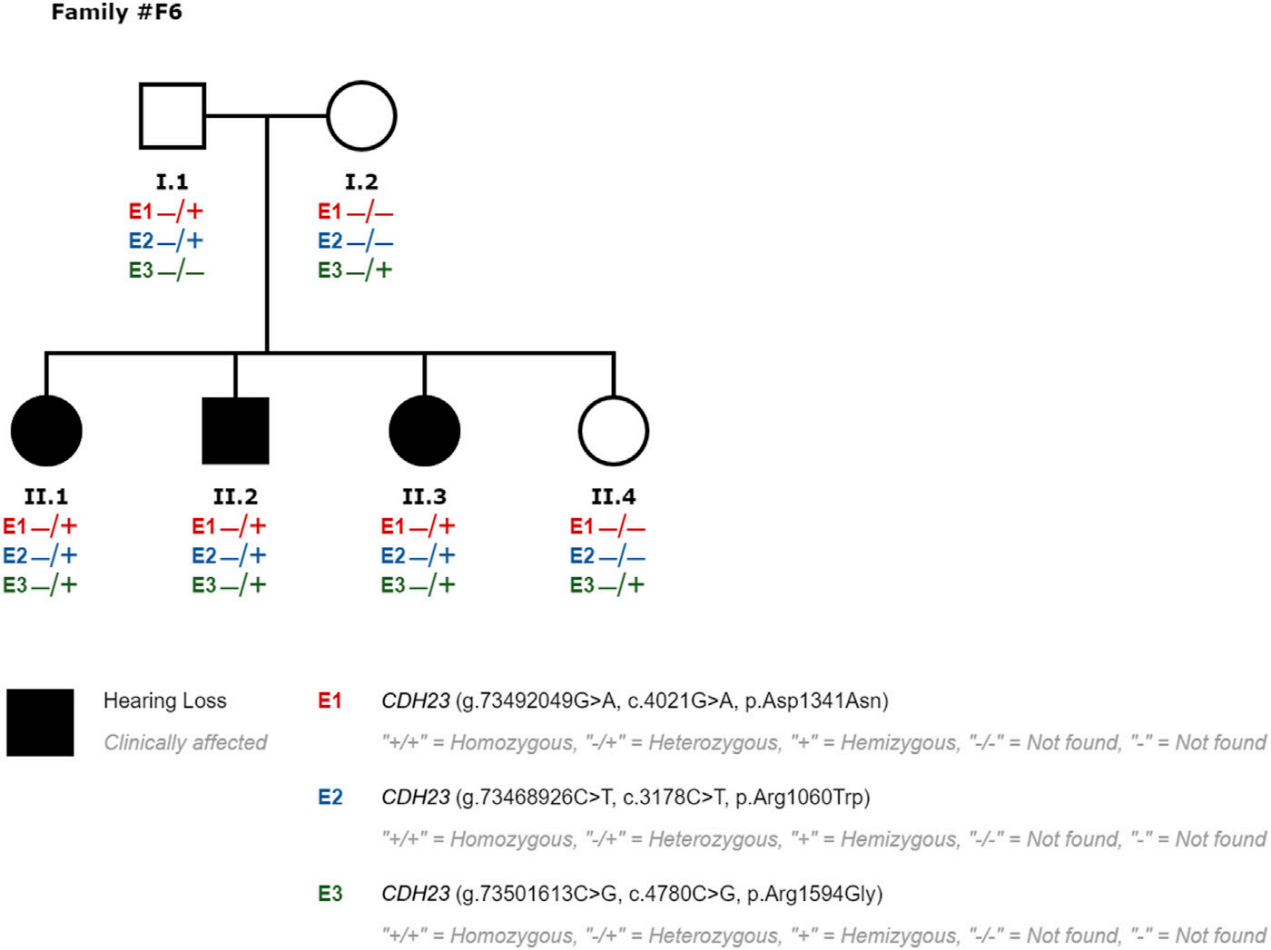
Segregation study performed in family #F6 for patients #P50 (II1), #P51 (II2), #P52 (II3) and hearing relatives to evaluate the significance of variants classified as VOUS. E3 variant was re-classified to likely pathogenic, responsible for SNHL in compound heterozygosity with E1 in this family.

We performed segregation studies to evaluate the significance of the identified variants. For example, in family #F6, an individual was revealed to carry three variants in the *CDH23* (NM_022124.6) gene: a likely pathogenic variant c.4021G>A p.(Asp1341Asn) and two VOUS: c.4780C>G p.(Arg1594Gly) and c.3178C>T p.(Arg1060Trp) (#P50 in Figure 3). Biallelic mutations in the cadherin-23 gene are responsible for both Usher syndrome 1D and recessive non-syndromic hearing loss (DFNB12). Two additional siblings of this family with SNHL were studied, and they were also found to have those same variants in *CDH23* (#P51 and #P52 in Figure 3). A family segregation study with both hearing parents as well as a hearing sibling (Figure 1) indicated that the alleles segregating in this family were c.4021G>A and c.3178C>T being in cis with c.4780C>G in trans; c.[4021G>A; c.3178C>T]; [ c.4780C>G]. Thus, c.4780C>G p.(Arg1594Gly) variant was re-classified from VOUS to likely pathogenic, raising diagnostic yield of the NGS custom panel up to 34% (52 out of 155). Usher syndrome type 1D could not be confirmed in this family inheriting *CDH23* biallelic pathogenic variants, also responsible for DFNB12.

Family #F7. Nine-year-old child with profound bilateral SNHL and no other relevant clinical features (#P92) in whom a previously not described heterozygous nonsense variant in the endothelin-B receptor gene, *EDNRB* (NM_000115.5), was identified and classified as likely pathogenic: c.65C>A p. (Ser22*). Homozygous (and very rarely heterozygous) loss of function mutations in *EDNRB* have previously been associated with Waardenburg syndrome type 4A (WS4A) whereas heterozygous mutations have been described in association with isolated Hirschsprung disease, and suggestively with Waardenburg syndrome type 2 (WS2) (Issa et al., 2017). A segregation study in both normal hearing parents was performed and demonstrated that the aforementioned *EDNRB* variant was inherited from the father, thus excluding an autosomal dominant *EDNRB* inheritance. This patient in family #F7 was therefore classified as a carrier with no genetic diagnosis for SNHL, and thus included in Group 3.

### 3.3 Group 3

Finally, in 43% of the studied SNHL individuals (67 out of 155) no pathogenic or likely pathogenic or suspicious VOUS that could explain the SNHL phenotype were identified. In this group, a single pathogenic or likely pathogenic variant in a gene previously associated with recessive SNHL (carriers) (Table 6), likely/benign or VOUS not consistent with SNHL, may have been identified.

**TABLE 6:** Patients from Group 3 in whom a single pathogenic or likely pathogenic variant in a gene previously associated with recessive SNHL were identified (carriers). Variants indicated with the symbol ▲ are novel variants.

Patient	Gene	Heterozygous pathogenic variant
#P89	*LRTOMT* (NM_001145308)	c.83G>C p.(Arg28Thr)▲
#P90	*MYO15A* (NM_016239)	c.10428dup p.(Tyr3477Leufs*41)▲
#P91	*GJB2* (NM_004004)	c.35delG p.(Gly12Valfs*2)
#P92	*EDNRB* (NM_000115)	c.65C>A p.(Ser22*)▲
#P93	*GJB2* (NM_004004)	c.101T>C p.(Met34Thr)
#P94	*LRP2* (NM_004525)	c.13821_13822del p.(Ser4608Cysfs*14)▲
#P95	*TMPRSS3* (NM_024022)	c.242C>G p.(Ser81*)
#P96	*HSD17B4* (NM_000414)	c.1369A>T p.(Asn457Tyr)
#P97	*CEACAM16* (NM_001039213)	c.859del p.(Gln287Argfs*34)
	*STRC* (NM_153700)	Potential deletion not confirmed
#P98	*OTOGL* (NM_173591)	c.948del p.(Leu316Phefs*6)
	*TMC1* (NM_138691)	c.884 + 1G>A p.?
#P99	*GJB2* (NM_004004)	c.269T>C p.(Leu90Pro)
	*TBC1D24* (NM_001199107)	c.724C>T p.(Arg242Cys)
#P100	*EPS8L2* (NM_022772)	c.477 + 1G>A p.?▲
#P101	*GJB2* (NM_004004)	c.35delG p.(Gly12Valfs*2)
#P102	*GJB2* (NM_004004)	c.269T>C p.(Leu90Pro)
#P103	*OTOA* (NM_144672)	c.2359G>T p.(Glu787*)
#P104	*OTOA* (NM_144672)	c.2359G>T p.(Glu787*)
#P105	*GJB2* (NM_004004)	c.35delG p.(Gly12Valfs*2)
#P106	*GJB2* (NM_004004)	c.439G>A p.(Glu147Lys)
#P107	*TMPRSS3* (NM_024022)	c.1276G>A p.(Ala426Thr)
#P108	*SLC26A4* (NM_000441)	c.1246A>C p.(Thr416Pro)
#P109	*GJB2* (NM_004004)	c.35delG p.(Gly12Valfs*2)
#P110	*GRXCR1* (NM_001080476)	c.710_711del p.(His237Argfs*42)▲

## 4 Discussion

Undiagnosed and untreated hearing loss gives rise to worse outcomes in language and cognitive abilities besides social, academic and behavioral problems that negatively affects the quality of life of people with hearing loss (Lieu et al., 2020). In the light of its critical relevance, an early diagnosis of hearing loss is imperative for early intervention, such as hearing devices placement recommendation, promoting language development and provision of genetic counseling. However, an accurate genetic diagnosis of hearing loss has been difficult to achieve due to the extremely high etiological, genetic and phenotypic heterogeneity in this complex trait.

In this regard, here we contribute to reinforce the utility of transferring NGS target panels to the clinical practice for early genetic diagnosis of hearing loss, which can boost the diagnostic yield and may help revealing a hidden syndrome. Indeed, NGS testing is included in the hearing loss diagnostic algorithm within the newest ACMG guidelines (Li et al., 2022). Therefore, an adequate implementation of genomic tools, like the one presented in this study, is key in order to boost diagnostic yield in SNHL.

In the light of this need, our NGS GHELP panel has been designed and has shown the capacity to identify different types of sequence variants known to be pathogenic including SNVs, indels and CNVs within both nuclear and mitochondrial genes. This entails some progress compared to other published NGS-based panels that may analyze a lower number of genes, lack mitochondrial genes in their design (García-García et al., 2020) or were validated in a smaller prescreened sample set (Cabanillas et al., 2018). Providing a genomic tool that allows for identification of genetic etiology of hearing loss with no need of additional tests favors its implementation in clinical practice and newborn screening programs since it reduces turnaround time and extra costs for those patients with no common variant causative of genetic hearing loss.

In this study, a genetic diagnosis was established in 34% of the studied individuals (52/155), with hearing loss as a part of a syndrome in at least five of them. Providing an accurate diagnosis in these patients through genetic testing and the consequent genetic counseling positively may impact children management and outcome, and benefit their families. Besides providing information about etiology regarding hearing loss, contributing to a better understanding of the nature of this trait, likelihood of recurrence might be estimated, several rehabilitation options may be considered, and referral to other specialties in case surveillance is recommended in previously undiagnosed syndromes is feasible. Moreover, although still in preclinical stages, a growing number of gene therapy approaches have arisen in recent years, as a potential treatment for hearing loss.

One of the first crucial approaches when implementing NGS in the clinical practice is to define the set of genes that the panel will target. Establishing a strong association between the targeted genes and SNHL was needed for the genes to be included in the design (Li et al., 2022), so incidental findings are avoided and proper variant interpretation and genetic counseling can be provided. Even if isolated hearing loss accounts for 70% of the cases, given that genes that give rise to syndromic hearing loss were also included in our design, “hidden syndromes” could be revealed as shown by at least five of the cases in Group 1. Furthermore, although most of the genetic alterations found are located in nuclear genes, a causative homoplasmic mutation in a mitochondrial gene was identified in family #F3. Since mitochondrial genome was included in the panel design, this variant could be identified and hence diagnosis could be established for this patient, who would have been missed otherwise. In addition, NGS approaches make it feasible to identify digenic inheritance cases as shown by family #F1 and non-classical variants in common genes as shown by family #F5.

Regarding different types of potentially causative alteration, not only is hearing loss caused by pathogenic SNV and indels, but CNVs have also been described as common contributors to this trait. Therefore, CNV analysis should be required when genetic testing for hearing loss is considered. Sufficient coverage analysis for the targeted region and bioinformatic analyses should also be guaranteed, and MLPA confirmation is recommended whenever possible. Patients in families #F1 and #F2 highlight the relevance of including not only SNVs and indels, but also CNVs assessment in the design of genomic tools like our NGS GHELP panel.

Surprisingly, no *STRC* deletion was identified, which is unexpected given the high prevalence of *STRC* alterations in Europe, accounting for 16% of the autosomal recessive cases in this population (del Castillo et al., 2022; Cabanillas et al., 2018). The possibility of false negatives due to low sensitivity was rejected, since validation samples with *STRC* deletion have been tested with this genomic tool, confirming its ability to accurately identify this alteration. Further or larger studies in Spanish population are needed in order to better understand what the recurrence of alterations in this gene is.

Besides performing a comprehensive test that allows for the identification of different types of alterations (SNvs, indels and CNVs) in both nuclear and mitochondrial genes, another cornerstone in NGS diagnostic pipelines is variant classification and clinical interpretation of the genetic findings by geneticists. In an effort to foster standardization and reduce discrepancies across laboratories, guidelines for variant interpretation have been developed. A Hearing Loss Variant Curation Expert Panel has been also created to provide expert guidance in the context of genomic interpretation of variants in hearing loss-related genes (Richards et al., 2015; Oza et al., 2018). Besides the existence of international expert panels and clinical guidelines, clinical laboratories are encouraged to participate in proficiency testing programs in order to better understand technical limitations of their own genomic tools.

A multidisciplinary approach when it comes to clinical interpretation of genomic results is mandatory. Cooperation between geneticists and physicians facilitated follow-up studies when needed, and eased access to clinical and family history information helping in the clinical interpretation process. In our laboratory at least two variant curators analyzed every case, so discrepancies in variant classification were discussed and agreed prior to final report writing. Periodic interdepartmental sessions with otorhinolaryngologists from Clínica Universidad de Navarra were arranged in order to assess genotype-phenotype correlation for every case. This approach can help increase the diagnostic yield, especially for those cases in which a genetic diagnosis cannot *a priori* be concluded. Benefits of this cooperation are brought to light especially in families in Group 2, in whom a follow up was needed in order to conclude with a diagnosis. Access to several members in family #F6 for segregation studies allowing pathogenicity assessment of identified variants was possible thanks to collaboration with clinicians. Further reviewing genotype-phenotype correlation for family #F7 with the clinicians, highlighted the fact that the proband in this family was born before 35 weeks of a twin pregnancy; thus, being a high-risk premature infant for having non-genetic hearing loss, consistent with the fact that no genetic cause could be elucidated.

Even though NGS approaches have been proven to reduce diagnostic odyssey in hearing loss, our GHELP panel has failed to identify a genetic cause for hearing loss in 67% of the patients (103 out of 155), even if the panel design allows for the detection of any variant type. The diagnostic yield reached in this study might be slightly lower than in other published works in the field due to several reasons. Firstly, a reason for having missed pathogenic variants responsible for hearing loss may be the panel design itself: causative genes that may not be included in the gene list; deep intronic variants which are not assessed, since only coding sequence ± 10 bp including splice sites are evaluated; and poor coverage due to insufficient read depth or misalignment may prevent variant identification throughout some coding regions. In addition to these technical limitations, it is possible that the criteria for including patients during the recruitment process were not adequately fulfilled. This oversight could have led to negative genetic testing results in patients experiencing hearing loss due to reasons other than genetics, as demonstrated by the aforementioned patient in family #F7. Within this specific group of patients, it is plausible that environmental factors played a significant role in contributing to their hearing impairments. Patients in whom there were other known risk factors for ototoxicity and no suspicion of genetic origin for hearing loss should have not been recruited. Furthermore, patients who had previously tested positive for common variants were not included in the study. Only patients who had tested negative or with not known previous genetic studies were recruited, which may have impacted the diagnostic yield. Further studies in larger unscreened populations are needed in order to have a deeper knowledge of the genetic landscape of hearing loss.

All things considered, here we contribute to endorse the clinical utility of comprehensive NGS panels in clinical practice and screening protocols for hearing loss, contributing to precision medicine in this condition. Furthermore, the authors reckon that the clinical data and observed genotype-phenotype correlations collected in this study will be immensely valuable as prognostic information for future newly diagnosed patients. The use of genomic tools that allow identification of causative pathogenic variants–SNVs/indels and CNVs—in both nuclear and mitochondrial genes in a suitable time span lessens the diagnostic odyssey in hearing loss, making its implementation in diagnostic algorithms worthwhile and warranted.

## Data Availability

The data presented in the study are deposited in the European Nucleotide Archive (ENA) repository, accession number PRJEB65602 (https://www.ebi.ac.uk/ena/browser/view/PRJEB65602).
